# Eliciting α7‐nAChR exerts cardioprotective effects on ischemic cardiomyopathy via activation of AMPK signalling

**DOI:** 10.1111/jcmm.14363

**Published:** 2019-05-06

**Authors:** Zhong‐Hao Lin, Yue‐Chun Li, Shu‐Jie Wu, Cheng Zheng, Yuan‐Zheng Lin, Hao Lian, Wei‐Qian Lin, Jia‐Feng Lin

**Affiliations:** ^1^ Department of Cardiology Second Affiliated Hospital and Yuying Children's Hospital of Wenzhou Medical University Wenzhou China

**Keywords:** Adenosine monophosphate‐activated protein kinase, cholinergic anti‐inflammatory pathway, inflammation, ischemic cardiomyopathy, post‐infarct remodeling

## Abstract

Our previous studies have reported that agonist of α7 nicotinic acetylcholine receptors prevented electrophysiological dysfunction of rats with ischaemic cardiomyopathy (ICM) by eliciting the cholinergic anti‐inflammatory pathway (CAP). Adenosine monophosphate‐activated protein kinase (AMPK) signalling is widely recognized exerting cardioprotective effect in various cardiomyopathy. Here, we aimed to investigate whether the protective effects of the CAP are associated with AMPK signalling in ICM. In vivo, coronary artery of rats was ligated for 4 weeks to induce the ICM and then treated with PNU‐282987 (CAP agonist) and BML‐275 dihydrochloride (AMPK antagonist) for 4 weeks. In vitro, primary macrophages harvested from rats were induced inflammation by Lipopolysaccharide (LPS) treatment and then treated with PNU‐282987 and BML‐275 dihydrochloride. In vivo, exciting CAP by PUN‐282987 elicited an activation of AMPK signalling, alleviated ventricular remodeling, modified the cardiac electrophysiological function, reduced the cardiac expression of collagens and inflammatory cytokines and maintained the integrity of ultrastructure in the ischemic heart. However, the benefits of CAP excitation were blunted by AMPK signaling antagonization. In vitro, excitation of the CAP was observed inhibiting the nuclear transfer of NF‐κB p65 of macrophages and promoting the transformation of Ly‐6C^high^ macrophages into Ly‐6C^low^ macrophages. However, inhibiting AMPK signalling by BML‐275 dihydrochloride reversed the CAP effect on LPS‐treated macrophages. Finally, our findings suggest that eliciting the CAP modulates the inflammatory response in ICM through regulating AMPK signalling.

## 1 INTRODUCTION

In recent years, with the development of new therapies, the mortality of acute myocardial infarction (MI) is decreasing, but it is still a major cause of disease‐related death globally.^1^ MI patients may ultimately develop ischaemic cardiomyopathy (ICM), whose 5‐year survival rate only 40%‐50%. Ventricular remodelling during ICM is a continuous, diverse and complex process, contributes to a deterioration of heart function and poor prognosis. Persistent and chronic inflammation plays an important role in the process.^2^

Our past studies have demonstrated that the cholinergic anti‐inflammatory pathway (CAP) has a protective role against fatal ventricular arrhythmias.^3^ The CAP, excited by α7 nicotinic acetylcholine receptors (α7‐nAChR), is part of a highly conserved endogenous mechanism regulates the magnitude of inflammatory responses and benefits a wide range of inflammatory diseases.^4^ Cholinergic stimulation blocks endothelial cell activation and leukocyte recruitment during inflammation, suppresses the production of proinflammatory cytokines,^5^ and natural killer cells are less efficient to trigger dendritic cells (DC) maturation under the activated‐CAP.^6^ Eliciting α7‐nAChR suppresses the inflammatory response in autoimmune encephalomyelitis,^7^ autoimmune myocarditis^8^ and the inflammation mediated by microglia in global ischaemia rats.^9^

Recent years, many researchers suggest that inflammation disrupts energy metabolism, wherein cytokines impede a range of metabolic pathways and cause further damage.^10^, ^11^, ^12^ Adenosine monophosphate‐activated protein kinase (AMPK) consists of three subunits, α, β and γ, which together form a functional enzyme that works as an energy sensor to provide metabolic adaptations. The activation of AMPK appears to have a protective effect on many heart diseases by adjusting energy metabolism and the immune system,^13^ such as attenuating the development of atherosclerosis by reducing Drp1‐mediated mitochondrial fission.^14^ And Krawczyk et al implicated AMPK as an inhibitor of DC activation that modulated inflammation response.^15^

The CAP and AMPK signalling both contribute a protective role in many involve conditions, especially in anti‐inflammation. Thus, we hypothesized that the protective effects of the CAP are associated with AMPK signalling in ICM and aimed to investigate this hypothesis.

## 2 METHODS

### 2.1 Animal preparation

All animal experiments met the criterion ratified by the Animal Ethics Committee of Wenzhou Medical University (Number wydw2014‐0058) and coincided with the Guide for the Care and Use of Laboratory Animals issued by the National Institutes of Health. Male 6‐month‐old SPF class Sprague‐Dawley rats, weighing 300‐320 g, were used in all experiments. Animals were purchased from the Shanghai Laboratory Animal Center of China and maintained in the Wenzhou Medical University Animal Facility under SPF class with controlled temperature (23 ± 2°C), humidity (45 ± 5%) and photoperiod (12‐hour light/dark cycle). Food and water were supplied by Wenzhou Medical University Animal Facilities.

### 2.2 ICM model

We made the model described by Jayasankar et al^16^ and conforms to the research standard described by G. Michael Felker et al.^17^ Surviving rats were measured by echocardiography to acquire left ventricular ejection fractions (LVEF). Rats conformed LVEF under 50% and/or a large akinetic aneurysm with hypokinesis at a non‐ischaemic area within the left ventricle (LV) were divided into three groups: ICM rats (ICM group), ICM rats with PNU‐282987 (PNU) treatment (ICM + PNU group) and ICM rats with PNU and BML‐275 dihydrochloride (BML) treatment (ICM + PNU+BML group). The administration dosages were as follows: 1 mg/kg PNU and 10 mg/kg BML. All drugs were dissolved in saline containing 0.2% dimethyl sulfoxide (DMSO) intraperitoneally injected every day. Sham‐operated rats and ischaemic cardiomyopathy rats were injected with saline containing 0.2% DMSO to exclude interference.

### 2.3 Echocardiography

Transthoracic echocardiography with an M‐mode transducer (12‐MHz phased‐array transducer; Sonos 5500, Philips USA, Bothell, WA) was performed by an experienced technician who was uninformed of the research groups. At the papillary muscle level, short‐axis views of M‐mode tracings were recorded through the anterior and posterior LV walls to measure LV end‐diastolic dimension (LVEDd), LV end‐systolic dimension (LVESd) and LV fraction shortening (LVFS). LVEF values were obtained according to the Simpson approach.

### 2.4 Electrocardiograph, left ventricular pressure and ventricular programmed electric stimulation

Rats were anaesthetized under isoflurane and an electrical physiology system (PowerLab 8/36; AD Instruments, Colorado Springs, CO) based on a computer was applied to measure the electrocardiograph (ECG) and pressure. The right carotid artery was isolated and pressure transducers were introduced into the left ventricle to measure heart rate (HR), systolic blood pressure (SBP), diastolic blood pressure (DBP), LV systolic pressure (LVSP) and LV end‐diastolic pressure (LVEDP). Biopotential leads were immobilized subcutaneously in the bottom left portion of the chest wall and the right pectoral muscle. LabChart 8 software (AD Instruments) was used to record the RR, PR, QRS and QT duration data. The Bazett formula was used in the calibration of the QTc for the heart rate. After ECG measurements, a thoracotomy was carried out on the rats to expose the heart. Two stimulating electrodes (made with silver metal with a diameter under 0.2 mm, except for the two contact points and covered by Teflon for insulation) were gently stuck into the heart and placed in contact with the apex of heart. The programmable‐stimulators were allowed to produce the pacing using PowerLab 8/36. The programmed electric stimulation (PES) protocol and scoring table were developed as described by Bélichard et al.^18^

**Figure 1:**
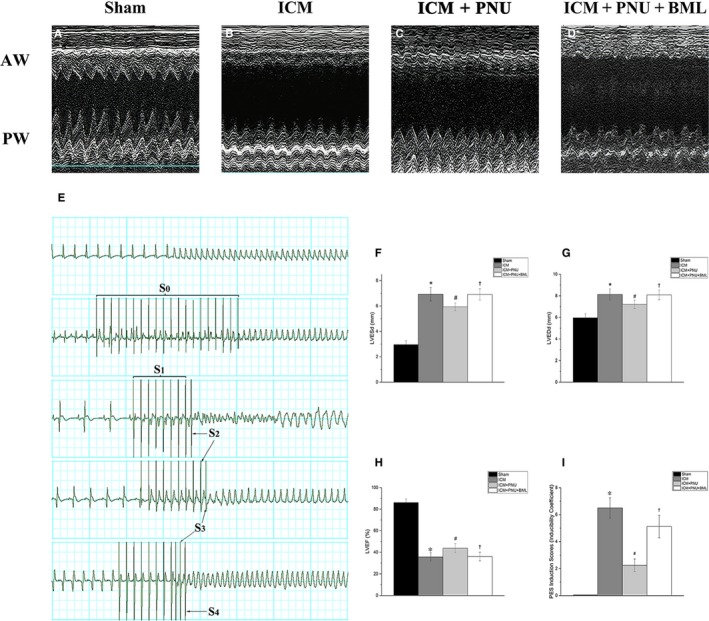
Suppressing Adenosine monophosphate‐activated protein kinase signalling blunted the improvement of cardiac function elicited by cholinergic anti‐inflammatory pathway. A‐D, The M‐mode echocardiograms of the short‐axis midventricular view of Sham‐operated rats (Sham, n = 8), 8‐wk after left anterior descending coronary artery ligation rats (ischaemic cardiomyopathy; ICM, n = 8), 4‐wk PNU treatment after 4‐wk left anterior descending coronary artery ligation rats (ICM + PNU, n = 8), ICM rats with 4‐wk PNU and BML (ICM + PNU + BML, n = 8). E, Typical examples of sustained ventricular arrhythmias induced by programmed electric stimulation (PES). S_0_, burst, 20 pace beats at a basic cycle length of 100 ms; S_1_, 8 paced beats at a basic cycle length of 100 ms; S2, S3 and S4 represent extra stimuli. F‐H, The difference of LVEDd, LVESd and LVEF among Sham group, ICM group, ICM + PNU group and ICM + PNU + BML group. The anterior wall (AW) of ICM rats performed distinct akinesis and thin, the posterior wall (PW) showed a certain degree of hypomotility and the left ventricular cavity expanded significantly. Compared with ICM group, PNU treatment reduced the LVEDd, LVESd, improved LVEF. LVEDd, LVESd and LVEF were similar among ICM group and ICM + PNU + BML group (all compared with ICM group, *P* > 0.05). I, PES induction scores, inducibility quotient of ventricular arrhythmias, n = 8 of each group. Data are present as means ± SD. Chi‐squared test and Kruskal‐Wallis *H* test were used to test the ranked data of inducibility of ventricular arrhythmias induced by PES. *P < 0.05 compared with Sham group. ^#^ *P* < 0.05 compared with ICM group. ^†^ *P* < 0.05 compared with ICM + PNU group

Under six beats with a self‐terminating ventricular tachyarrhythmia was considered ‘non‐inducible’. Ventricular tachyarrhythmia lasted >15 beats was ‘sustained group’, while a ventricular tachyarrhythmia that lasted ≤15 beats was ‘non‐sustained group’. The modes of PES (Figure 1E) were: S_0_ consisted of 20 pace beats at a basic cycle length of 100 ms; S_1_ consisted of a premature stimulus that was applied to identify the effective refractory period after eight paced beats that were uniformly distributed at a basic cycle length of 100 ms; S_2_, S_3_ and S_4_ consisted of S_1_ with 1×, 2× and 3× extra stimuli.

The PES scoring table was: 0, ‘non‐inducible’ or no premature ventricular beats; 1, non‐sustained ventricular tachyarrhythmias induced by S_4_; 2, sustained ventricular tachyarrhythmias induced by S_4_; 3, non‐sustained ventricular tachyarrhythmias induced by S_3_; 4, sustained ventricular tachyarrhythmias induced by S_3_; 5, non‐sustained ventricular tachyarrhythmias induced by S_2_; 6, sustained ventricular tachyarrhythmias induced by S_2_; 7, ventricular tachyarrhythmias induced by S_0_; 8, non‐induced sustained ventricular tachyarrhythmias, cardiac arrest or sudden death appeared before PES or during the preparation.^19^

### 2.5 Cell culture

1. Macrophages: Primary peritoneal macrophages were harvested from healthy male Sprague‐Dawley rats (SPSS class, 300‐320 g, 6 months old) using a protocol similar to Xia Zhang et al.^20^
2. Inflammatory model: Cells that were pretreated with PNU (50 nmol/L) and BML (10 μmol/L) were stimulated by lipopolysaccharide (LPS, 100 ng/mL). BML was introduced 1 hour before PNU. Unless otherwise specified, the concentrations of PNU and BML used in cellular experiments were similar above.

### 2.6 The extent of fibrosis

The middle 1/3 of the left ventricle (the LV middle ring) was embedded in paraffin, sectioned into 5 μm‐thick slices and the Masson's trichrome‐stained dyeing method was applied. The degree of fibrosis was expressed as the mean percentage of the total border zone and the protein expression level of collagen I and III.

### 2.7 Transmission electron microscopy

Fresh tissue pieces (approximately 1 mm × 1 mm × 1 mm) from the mid‐myocardium of middle 1/3 of the LV of the infarct border zone were fixed in glutaraldehyde and osmium tetroxide, embedded in epoxy resin. An experienced technician who was ignorant about the groups processed the treated samples into ultrathin sections and made all measurements.

### 2.8 Immunocytochemistry

Fresh tissue samples embedded in OCT Compound (−30°C) were sectioned into 5 μm‐thick slices. Thawed slides were fixed in 4°C acetones for 10 minutes. Cell samples were fixed with 4% paraformaldehyde for 15 minutes and permeabilized with 0.5% Triton X‐100 for 10 minutes. Slides were washed in 1× PBS three times after each step. Sufficient reagent was used to cover the specimen and the reagents were removed after each step using suction. The specimens were first incubated with 10% blocking serum in PBS at room temperature for 30 minutes and then washed off with PBS for 5 minutes each. Next, the specimens were incubated with primary antibodies overnight at 4°C, and then the antibodies were washed off with PBS for 5 minutes each. The secondary antibodies were then added and the specimens were incubated at room temperature for 60 minutes. Washed off with PBS for 5 minutes each, and then the specimens were incubated with 4,6‐diamidino‐2‐phenylindole dihydrochloride (DAPI, Beyotime, Shanghai, China, C1005), for 10 minutes at room temperature. Finally, washed off with PBS for 5 minutes each and the coverslip mounted with an Anti‐Fluorescence Quenching Sealant (Beyotime, Shanghai, China, P0126). The samples were stored in a covered opaque dark wet box and examined using a fluorescence microscope with the appropriate filters (DP72, Olympus, Waltham, MA). All steps were performed in the dark, Antibody diluent was 1% blocking serum in PBS. The primary antibodies included the Connexin 43 Antibody (Cx‐43, 1:200; CST, #3512) and nuclear factor‐κB p65 (NF‐κB p65, 1:200, CST, D14E12). The secondary antibody was fluorescein isothiocyanate (FITC, 1:500; Cytoskeleton, 33107ES60). And rhodamine phalloidin (1:200; Cytoskeleton, 33209ES60).

### 2.9 Western blotting

Protein was extracted from the treated primary macrophages and the marginal area around the infarct area. RIPA lysate (ThermoFisher, 89900), protease inhibitors (Beyotime, Jiangsu, China, ST506‐2) and phosphatase inhibitors (Applygen Technologies, Beijing, China, P1260) were added in to lyse the cells or tissues. A BCA protein assay kit (ThermoFisher, 23235) was used to measure the protein concentration. Then the protein was mixed with double distilled H_2_O and 5× loading buffer to a final volume ratio of loading buffer to sample of 1:5 to finally get the mixture sample. The mixture samples should have the same protein concentration, and the protein should not higher than 80% volume of homologous mixture sample. After that, boiled the mixture at 100°C for 10 minutes. Next, each sample was separated by 10% SDS‐PAGE and transferred to a polyvinylidene difluoride membrane. The membranes were blocked in 5% milk (Skim Milk; BD, Difco^TM^). 1× mixture of Tris‐buffered saline and Polysorbate 20 was applied to wash the membranes (5 mintues, three times). After being incubated in specific primary (12 hours, 4°C) and secondary antibodies (1‐hour, room temperature), the amount of protein on the membranes were measured by enhanced chemiluminescence detection system (Millipore, Billerica, MA). The antibodies against peroxisome proliferator‐activated receptor gamma coactivator 1‐alpha (PGC‐1α, 3G6), AMP‐activated protein kinase‐alpha (AMPKα, D5A2), phospho‐AMPKα (Thr172, 40H9), Acetyl‐CoA Carboxylase (ACC, C83B10), phospho‐Acetyl‐CoA Carboxylase (Ser79, D7D11), Jak2 (D2E12), phospho‐Jak2 (Tyr1008, D4A8), Stat3 (D3Z2G), phospho‐Stat3 (Tyr705, D3A7), NF‐κB p65, phospho‐ NF‐κB p65 (Ser536, 93H1), Cx‐43 and GAPDH were purchased from CST. Antibodies against monocyte chemoattractant protein‐1 (MCP‐1, ab25124), intercellular adhesion molecule‐1 (ICAM‐1, ab171123), tumour necrosis factor alpha (TNFα, ab220210), interleukin 6 (IL‐6, ab9324) and IL‐1β (ab9722) were from Abcam. Collagen I (14695‐1‐AP) and Collagen III (22734‐1‐AP) antibodies were from Proteintech.

### 2.10 Flow cytometry

Primary macrophages were harvested and incubated at 37°C, 5% CO_2 _in 1× RPMI medium 1640 basic supplemented with 10% foetal bovine serum. The medium was changed 3 hours after cell plating to remove non‐adherent cells. To exclude the interference, continue incubated 12 hours, replaced the medium again. Then treated with PNU and BML for 12 hours and stimulated with LPS. Harvested the cells by 0.25% EDTA (3 minutes at 37°C, 5% CO_2_, 95% humidity). Taken a small sample to perform a cell count. And then pipetted off the cells and centrifuged for 5 minutes at 194 *g*, the supernatant was discarded, and the pellet was resuspended in enough 1× PBS to have a final cell concentration of 10 million cells/mL. Then, 100 µL of prepared cell suspension was aspirated into a labelled tube and 1 µg of primary/fluorochrome‐conjugated antibodies were added. The samples were vortexed and incubated for 30 minutes in the dark at 4°C, or in a covered ice bucket. Next, 2 mL of 1× PBS was added to each tube and the samples were centrifuged for 5 minutes at 778 *g* to wash off the excess antibody following staining. The supernatant was aspirated, being careful not to disturb the pellet. Indirect staining requires fluorochrome‐conjugated secondary antibodies. A total of 100 µL of 1× PBS was added to resuspend the binding protein/antibodies, and thereafter 1 µg was added. The samples were vortexed and incubated for 30 minutes in the dark at 4°C or on ice, and the excess antibody was washed off following staining. Samples were then centrifuged for 5 minutes at 778 *g*. The supernatant was aspirated and 500 µL of 1× PBS or 500 µL of 1% paraformaldehyde was added to resuspend the pellet for detection or storage, respectively. The antibodies included Ly‐6C (G‐3, sc‐271811), and F4/80 (C‐7, sc‐377009) were purchased from Santa Cruz. Anti‐CD11b/c antibodies were purchase from abcam (OX42, ab1211) and MultiSciences (70‐AR011BC04‐100). Goat Anti‐Mouse IgG/APC (bs‐0296G‐APC) was from Bioss ANTIBODIES. The machine is CytoFLEX (BECKMAN COULTER, Model A00‐1‐1102).

### 2.11 Statistical methods

SPSS 22 software (Unicom, Mosson Hills, CA) was used for statistical analyses. A value of *P* < 0.05 was considered significant. The data are expressed as the means ± SD. For data with variance homogeneity, all outcomes among groups were compared using a one‐way ANOVA, followed by the Dunnett multiple‐comparison test. Chi‐squared tests and Kruskal‐Wallis *H* tests were used on the ranked data of inducibility of ventricular arrhythmias induced by PES.

## 3 RESULTS

### 3.1 Suppressing AMPK signalling blunted the improvement of cardiac function and PES‐induced ventricular tachyarrhythmia elicited by the CAP

In Figure 1, the 4‐week PNU treatment improved LVEDd, LVESd and LVEF. The values for the ICM group compared to the ICM + PNU group were 8.13 ± 0.52 vs 7.21 ± 0.38 for LVEDd (*P* < 0.05), 6.93 ± 0.53 vs 5.94 ± 0.31 for LVESd (*P* < 0.05), and 35.73 ± 3.83 vs 43.84 ± 4.24 for LVEF (*P* < 0.05). The ICM + PNU+BML group was similar to the ICM group, with values of 8.13 ± 0.52 vs 8.14 ± 0.61 for LVEDd (*P* > 0.05), 6.93 ± 0.53 vs 6.93 ± 0.49 for LVESd (*P* > 0.05), 35.73 ± 3.83 vs 37.78 ± 4.38 for LVEF (*P* > 0.05) and 14.83 ± 1.81 vs 15.06 ± 1.97 for LVFS (*P* > 0.05) for the ICM group vs the ICM + PNU + BML group, respectively. ICM rats had a higher ventricular arrhythmia quotient induced by PES (ICM group vs the Sham group, *P* < 0.05), and PNU‐treated rats had lower quotients (compared to the ICM group, *P* < 0.05). The effects of PNU treatment were inhibited by BML (ICM + PNU + BML group vs ICM + PNU group, *P* < 0.05; ICM + PNU + BML group vs ICM group, *P* > 0.05). Table 1 provides further proof of the positive effects of PNU treatment and the harmful influence of AMPKα inhibition. The ICM group displayed prolonged QTc, lower SBP, DBP and LVSP and higher LVEDP (compared to Sham group, *P* < 0.05). Compared to ICM group, PNU treatment improved QTc, SBP, LVSP and LVEDP (*P* < 0.05). The ICM + PNU + BML group showed prolonged QTc, lower SBP, DBP and LVSP and higher LVEDP (compared to the ICM + PNU group, *P* < 0.05), which was similar to ICM group. The RR interval, PR interval, duration of P‐wave and duration of QRS‐wave were similar among the groups (*P* > 0.05).

**Table 1:** Suppressing AMPK signalling blunted the improvement of BP, left ventricular pressure and QTc elicited by CAP

Group	RR (ms)	P (ms)	PR (ms)	QRS (ms)	QTc (ms)	HR (bpm)	SBP (mm Hg)	DBP (mm Hg)	LVSP (mm Hg)	LVEDP (mm Hg)
Sham	197.42 ± 20.82	50.56 ± 0.91	17.60 ± 1.56	25.38 ± 3.92	133.92 ± 3.42	357 ± 11	100.44 ± 2.78	75.19 ± 3.00	137.48 ± 1.30	3.13 ± 0.57
ICM	196.21 ± 20.64	50.60 ± 1.29	17.93 ± 1.36	24.72 ± 1.51	172.13 ± 6.97*	350 ± 21	88.97 ± 3.13*	65.76 ± 3.11*	92.49 ± 1.53*	7.14 ± 0.75*
ICM + PNU	192.19 ± 14.85	50.22 ± 3.07	18.01 ± 1.39	25.43 ± 2.66	153.03 ± 6.69^#^	355 ± 27	92.38 ± 2.84^#^	66.50 ± 2.45	111.01 ± 2.41^#^	6.40 ± 1.79^#^
ICM + PNU + BML	192.25 ± 21.96	50.58 ± 2.28	18.24 ± 0.92	24.98 ± 1.42	161.53 ± 8.90^†^	349 ± 23	89.70 ± 1.88^†^	65.16 ± 2.29^†^	98.40 ± 3.23^†^	7.17 ± 1.28^†^

Note

n = 8 of each group. Data are present as means ± SD.

Abbreviations: AMPK, Adenosine monophosphate‐activated protein kinase; CAP, cholinergic anti‐inflammatory pathway; DBP, diastolic blood pressure; HR, heart rate; ICM, ischemic cardiomyopathy; LVEDP, left ventricular end‐diastolic pressure; LVSP, left ventricular systolic pressure; P, P‐wave duration; PR, P‐R interval; QRS, QRS‐wave interval; QTc, heart rate‐corrected QT interval; RR, R‐R interval; SBP, systolic blood pressure.

* *P* < 0.05 compared with Sham group.

^#^ *P* < 0.05 compared with ICM group.

^†^ *P* < 0.05 compared with ICM + PNU group.

### 3.2 Suppressing AMPK signalling blunted the decrease in fibrosis and proinflammatory cytokine production elicited by the CAP in the infarcted border zone

**Figure 2:**
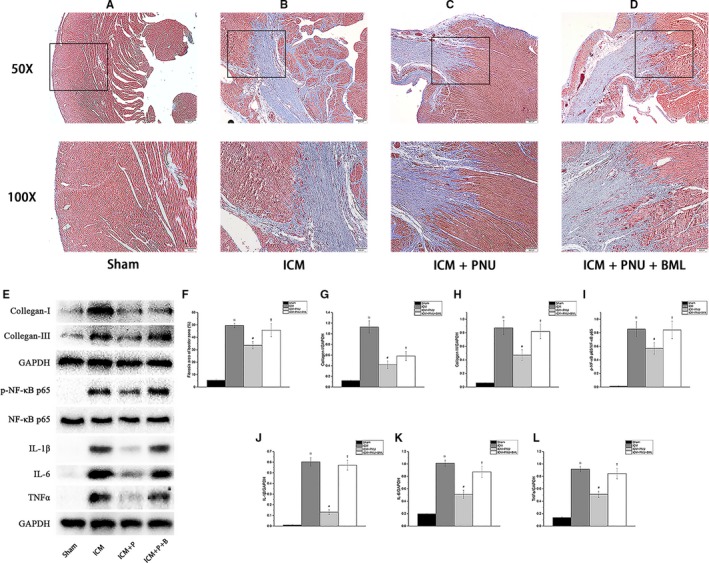
Suppressing Adenosine monophosphate‐activated protein kinase signalling blunted the decreasing of fibrosis and proinflammatory cytokines production of infarcted border zone elicited by cholinergic anti‐inflammatory pathway. A‐D, Fibrosis area of border zone (%) among 4 groups (A, Sham group, n = 8; B, ischaemic cardiomyopathy; ICM group, n = 8; C, ICM + PNU group, n = 8; D, ICM + PNU + BML group, n = 8; upper magnification ×50; lower magnification ×100). E, The Western blotting results, n = 8 for each group; ICM + P, ICM + PNU group; ICM + P + B, ICM + PNU + BML group. F‐L, Analysis results of A‐E. Compared with Sham group, fibrosis in border zone, collagen synthesis/release and inflammatory response were significantly increased in ICM group. PNU treatment showed an evident improvement of these targets. Addition of BML attenuated these effects of PNU treatment. Data are present as means ± SD. *P < 0.05 compared with Sham group. ^#^ *P* < 0.05 compared with ICM group. ^†^ *P* < 0.05 compared with ICM + PNU group

The area of fibrosis in the infarcted border zone was shown by Masson's trichrome‐staining, and the collagen synthesis/release and proinflammatory cytokine production were displayed by western blotting (Figure 2). Compared to the Sham group, the area of fibrosis in the ICM group was significantly increased (ICM group vs the Sham group, *P* < 0.05), as well as the synthesis/release of collagen (ICM group vs the Sham group, Collagen I/GAPDH, *P* < 0.05; Collagen III/GAPDH, *P* < 0.05) and the proinflammatory cytokine production (ICM group vs the Sham group, p‐NF‐κB p65/NF‐κB p65, *P* < 0.05; TNFα, *P* < 0.05; IL‐6, *P* < 0.05; IL‐1β, *P* < 0.05). PNU treatment improved collagen synthesis/release, decreased proinflammatory cytokine production and decreased the area of fibrous (all compared to the ICM group, *P* < 0.05). The modified effects of PNU treatment were inhibited by BML (all compared to the ICM + PNU group, *P* < 0.05).

### 3.3 Suppressing AMPK signalling blunted the preservation of Connexin‐43 (Cx‐43) elicited by the CAP

**Figure 3:**
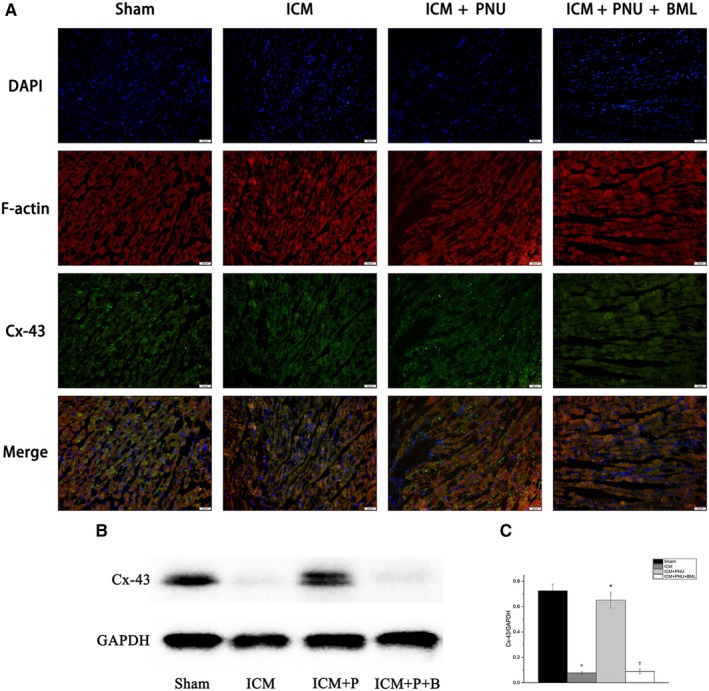
Suppressing Adenosine monophosphate‐activated protein kinase signalling blunted the protecting of Connexin‐43 (Cx‐43) elicited by cholinergic anti‐inflammatory pathway. A, Immunofluorescence images of Cx‐43 and F‐actin from left ventricles of rats, n = 8 of each group; DAPI, 4′,6‐diamidino‐2‐phenylindole; Cx‐43, connexion 43; magnification ×200. The positive immuno‐active signals of Cx‐43 (green) were accumulated in intercellular positions. The increased and non‐uniform of F‐Actin (red) represent cardiomyocyte hypertrophy and damage. B‐D, Results of Western blotting of Cx‐43, n = 8 of each group. Data are present as means ± SD. *P < 0.05 compared with Sham group. ^#^ *P* < 0.05 compared with ischemic cardiomyopathy (ICM) group. ^†^ *P* < 0.05 compared with ICM + PNU group

In Figure 3, the ICM group had a significant decrease in the expression of the protein Cx‐43 when compared with Sham group. And the F‐Actin was evidently increased and non‐uniform in the ICM group compared to the Sham group. The distribution of Cx‐43 in the ICM + PNU group tended to be denser and was much greater than in the ICM group. BML treatment prevented this. Similar changes were observed by Western blot (Cx‐43, ICM group vs the Sham group, *P* < 0.05; ICM + PNU group vs ICM group, *P* < 0.05; ICM + PNU + BML group vs ICM + PNU group, *P* < 0.05; ICM + PNU + BML group vs ICM group, *P* > 0.05). The inhibition of IL‐1β after MI can increase the expression of Cx43^6^ and the Western blot results matched this condition (Figure 2).

### 3.4 Suppressing AMPK signalling blunted the ischaemic myocardium ultrastructure improvement elicited by the CAP

**Figure 4:**
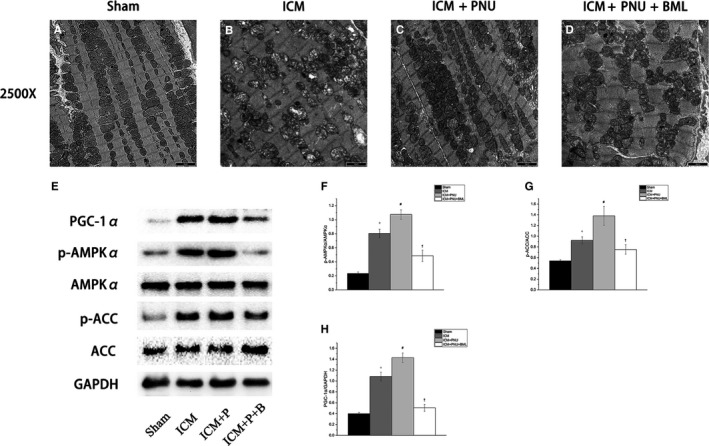
Suppressing Adenosine monophosphate‐activated protein kinase (AMPK) signalling blunted the improvement of ultrastructure of ischaemic myocardium elicited by cholinergic anti‐inflammatory pathway. A‐D, Representative transmission electron microscope images from rat left ventricles (A, Sham group, n = 8; B, ischaemic cardiomyopathy [ICM] group, n = 8; C, ICM + PNU group, n = 8; D, ICM + PNU + BML group, n = 8; magnification ×2500). E‐H, Western blotting results, n = 8 for each group; ICM + P, ICM + PNU; ICM + P + B, ICM + PNU + BML; PGC‐1α, peroxisome proliferator‐activated receptor gamma coactivator 1‐alpha; p‐AMPKα, phosphorylated Adenosine monophosphate‐activated protein kinase; p‐ACC, phosphorylated Acetyl‐CoA Carboxylase. Data are present as means ± SD. *P < 0.05 compared with Sham group. ^#^ *P* < 0.05 compared with ICM group. ^†^ *P* < 0.05 compared with ICM + PNU group

In Figure 4, transmission electron microscopy was used to observe the ultrastructure of the ischaemic myocardium. Compared to the Sham group, the sarcomeres around the border zone were ruined and the mitochondria became swollen and destroyed in the ICM group. PNU treatment improved these conditions. BML treatment showed the opposite tendency when compared to the PNU treatment. The Western blot results provided further proof. The ICM group showed higher levels of phosphorylated AMPKα/ACC and PGC‐1α (ICM group compared to the Sham group; p‐AMPKα, *P* < 0.05; p‐ACC, *P* < 0.05; PGC‐1α, *P* < 0.05). PNU treatment enhanced the expression of these proteins (ICM + PNU group compared to the ICM group; p‐AMPKα, *P* < 0.05; p‐ACC, *P* < 0.05; PGC‐1α, *P* < 0.05). The addition of BML induced the opposite expression (ICM + PNU + BML group vs ICM + PNU group; p‐AMPKα, *P* < 0.05; p‐ACC, *P* < 0.05; PGC‐1α, *P* < 0.05).

### 3.5 Suppressing AMPK signalling blunted the inhibition of the nuclear transfer of NF‐κB p65 elicited by the CAP

**Figure 5:**
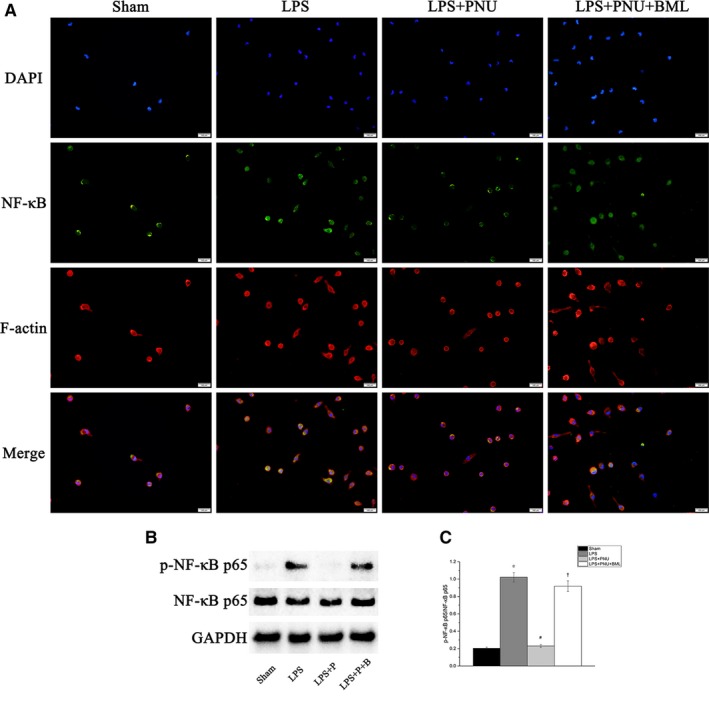
Suppressing It is Adenosine monophosphate‐activated protein kinase signalling blunted the inhibition of nuclear transfer of NF‐κB p65 elicited by cholinergic anti‐inflammatory pathway. Sham, Sham group, n = 8; LPS, LPS group, n = 8; LPS + P, LPS + PNU group, n = 8; LPS + P + B, LPS + PNU + BML group, n = 8; LPS, lipopolysaccharide. Sham group, cells without any special treatment. All groups maintained in common incubator (37°C, 95% humidity, 5% CO_2_). A, Representative immunofluorescence images of NF‐κB activation in LPS‐stimulated primary macrophages; magnification ×400. B‐C, Western blotting results of cell culture. Data are present as means ± SD. *P < 0.05 compared with Sham. ^#^ *P* < 0.05 compared with LPS group. ^†^ *P* < 0.05 compared with LPS + PNU group

NF‐κB is a classical transcriptional regulator influence the inflammatory response. In Figure 5, the LPS group had substantially increased phosphorylation of NF‐κB p65. PNU treatment inhibited the enhanced NF‐κB p65 nuclear transfer. BML treatment blocked the inhibition of the PNU treatment. The Western blot results provided further proof of these observations (p‐NF‐κB p65, LPS group vs Sham group, *P* < 0.05; LPS + PNU group vs LPS group, *P* < 0.05; LPS + PNU + BML group vs LPS + PNU group, *P* < 0.05).

### 3.6 Suppressing AMPK signalling blunted the effect of the CAP on inflammatory and reparative macrophages

**Figure 6:**
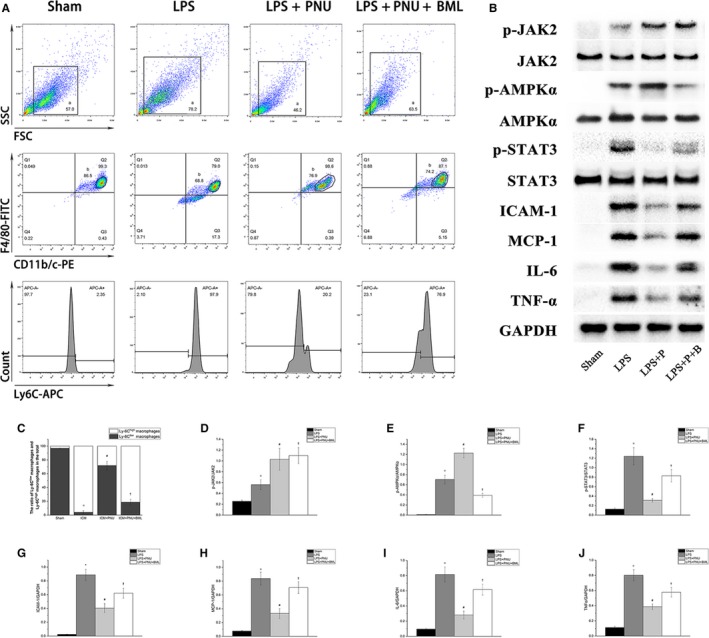
Suppressing Adenosine monophosphate‐activated protein kinase signalling blunted the effect of cholinergic anti‐inflammatory pathway on the inflammatory and reparative Macrophages. Sham, Sham group, n = 8; LPS, LPS group, n = 8; LPS + P, LPS + PNU group, n = 8; LPS + P + B, LPS + PNU + BML group, n = 8; LPS, lipopolysaccharide. Sham group, cells without any special treatment. All groups maintained in common incubator (37°C, 95% humidity, 5% CO_2_). A, Representative flow cytometry images of treated primary macrophages. B, Western blotting results. C‐J, Analysis results of A and B

Ly‐6C^high^ and Ly‐6C^low^ macrophages are two subsets of macrophages. The Ly‐6C^high^ macrophages are inflammatory macrophages that appear earlier at injured sites, phagocytose dead tissue and release inflammatory mediators. The Ly‐6C^low^ macrophages are reparative macrophages that support angiogenesis and the synthesis of the extracellular matrix. MCP‐1 and ICAM‐1 are involved in the adhesion and migration of leukocytes and recruit leukocytes to sites of inflammation produced by tissue injury or infection. In Figure 6, the LPS group had higher levels of Ly‐6C^high^ macrophages, increased expression of MCP‐1, ICAM‐1 and other inflammatory cytokines, such as IL‐6 and TNFα (LPS group vs the Sham group, *P* < 0.05). Eliciting the CAP transformed the Ly‐6C^high^ macrophages into Ly‐6C^low^ macrophages and significantly reduced the expression of MCP‐1, ICAM‐1 and inflammatory cytokines (all compared to the LPS group, *P* < 0.05). Inhibition of AMPK blunted the anti‐inflammatory expression and the transformation effect (LPS + PNU + BML group compared to the LPS + PNU group, *P* < 0.05).

## 4 DISCUSSION

In this study, we chose a specific α7‐nAChR agonist to investigate how eliciting the CAP would improve the cardiac function of ICM and to understand the potential mechanism behind it. We found that compared to rats subjected to a sham operation, ICM rats showed significant fibrosis and ongoing inflammation in the infarct border zone, as well as a lower ejection fraction, lower blood pressure, lower left ventricular pressure and higher arrhythmogenic properties. The ultrastructure was observed by transmission electron microscopy; the mitochondria and myocardial sarcomeres were swollen and destroyed in the ICM group. PNU treatment improved these harmful variations and increased the levels of the gap junction protein, Cx‐43. The addition of BML inhibited the above changes. The increased expression of phospho‐AMPKα and the results from experiments inhibiting AMPKα phosphorylation suggested that eliciting the CAP exerts anti‐inflammatory, anti‐fibrotic and anti‐arrhythmic effects via AMPK signalling.

To explain the above findings, we hypothesize that left anterior descending coronary artery infarct activates the immune system to recruit proinflammatory macrophages, causing a regional accumulation of cytokines at the injury site that disrupts energy metabolism, influences angiogenesis, promotes autophagy and facilitates the generation of fibrotic tissue.^13^, ^21^, ^22^, ^23^ ICM is the end stage of MI, in which a half‐hypoxic and half‐ischemic environment at the infarct border zone creates a special pathological condition that causes aseptic chronic inflammation, chronic fibrosis and ventricular remodelling. Several studies revealed that post‐infarct remodelling was associated with the persisting low‐level inflammatory response and tissue fibrosis, modulation of the inflammatory and fibrotic responses could, to some extent, reverse the process of post‐infarct remodeling.^2^, ^24^ The most ventricular cardiomyocytes of infarct zone died, but the Purkinje fibers survived and the new generation of fibrosis layer with prolonged action potentials and enhanced automaticity.^25^, ^26^ Ventricular remodelling of the infarcted heart causes persistent electrical activity and impulse propagation, increase the sudden death, which has been reported to be closely related to changes in gap junctions, especially the Cx‐43 protein.^27^, ^28^ Genetically engineered Cx43‐deficient mice are markedly more susceptible to ischaemia‐induced ventricular tachycardia.^29^, ^30^, ^31^ Our study supported that activating AMPK signalling via the CAP can suppress the inflammation response and increase the expression of Cx‐43, inhibition of the AMPK attenuated the Cx‐43 expression. The decrease in IL‐1β release inhibited the damage of Cx‐43.^32^ The inhibition of inflammation and the functional preservation of Cx43 would play an irreplaceable role in protective properties during ICM.

Few treatment options are available to improve the development and progression of heart failure and fatal ventricular arrhythmia induced by cardiac ischaemia that makes the clinical treatment of ICM a challenging problem. The heart requires unremitting energy production to match functional demands. The infarction disrupts the blood supply to the heart, resulting in a mismatch between the energy demand and energy supply of the heart and causing the death of cardiomyocytes and heart failure, which is closely related to fatal ventricular arrhythmias and sudden cardiac death.^33^ While heart failure initially involves the myocardium and results in decreased cardiac performance, it rapidly affects multiple organs including the neurohormonal, circulatory and renal systems. Indeed, patients with heart failure have chronic activation of the sympathetic nervous system, which results in a maladaptive attempt to improve cardiac function.^34^ Now researchers have realized that the essence of heart failure is ‘energy starvation’,^35^ regardless of the absence of blood supply or the disorder to energy metabolism of myocytes. The cytokines released by the inflammation of the ischaemic area impede a range of metabolic pathways and disrupt energy metabolism.^10^, ^11^, ^12^ The AMPK pathway is an important integrator of signals that controls energy balance through the regulation of multiple biochemical pathways in all eukaryotes, and the AMPKα/ACC pathway is important for energy metabolism adjustments in vivo.^13^ Phosphorylated AMPKα enhances mitochondrial biogenesis through its effects on the transcriptional coactivator, PGC‐1α.^36^ PGC‐1α is increased in response to the activation of AMPK and is reduced in AMPKα2 null mice.^37^ Our study shows that eliciting α7‐nAChR significantly increased the phosphorylation of AMPKα. Increased phosphorylated AMPKα/ACC can facilitate lipid energy metabolism, improve the survival environment and provide more energy to cardiomyocytes. The arrhythmia score of PES‐induced ventricular arrhythmia was increased when AMPK signalling was inhibited, and proinflammatory cytokines and collagen synthesis/release were increased as well. The inhibition effect of BML in the phosphorylation of AMPK has been reported,^38^ but its underlying mechanisms are unclear. Otherwise, the increased phosphorylation AMPK play a protective effect in functional preservation of Cx43,^39^ it may because of the change in AMP/ATP ratio and Ca^2+^.^40^ Although the AMPK knock down model is more significant, missense mutations or subunit‐deficient AMPK cause hypertrophic cardiomyopathies, heart failure, atrial fibrillation, ventricular arrhythmia and other metabolic diseases.^41^ It can't match the clinical diagnostic standard of ICM and will interfere with our observation of ICM.

Ly‐6C^high^ and Ly‐6C^low^ macrophages are two subsets of macrophages. Ly‐6C^high^ macrophages are inflammatory macrophages appear earlier at injured sites, phagocytose dead tissue and release inflammatory mediators. Ly‐6C^low^ macrophages are reparative macrophages support angiogenesis and the synthesis of the extracellular matrix. A series studies have indicated that monocytes/macrophages not only act as a defense against harmful factors but also help to keep the body healthy. The exhaustion of tissue‐resident macrophages in heart could cause the development of an atrioventricular block.^42^ These macrophages also express abundant Cx‐43. Tissue‐resident macrophages are relatively independent of monocytes/macrophages recruited from circulation and have longer lives and potentially self‐renewal abilities. Tissue‐resident macrophages mostly perform partial self‐renewal with a long cycle, which is different from the renewal of monocytes/macrophages in circulation.^43^ But the huge requirement for macrophages during pathological conditions disrupts the isolation between these two types, plenty monocytes/macrophages are recruited and the transformation is available in order to supply the expend of macrophages in the injured tissue.^44^ Moreover, the local injection of tolerogenic dendritic cells can induce the immune system, causes the migration of inflammatory‐repaired macrophages to the infarct heart, change the immune environment and these cells have been shown to play a positive role in the rebuilding of the necrotic myocardium.^45^ Ly‐6C^low^ macrophages also play a crucial role in the pathogenesis and progression of the healing myocardium.^22^, ^46^ Regulating the activation of proinflammatory macrophages post‐myocardial infarction improves cardiac remodelling. Besides, the levels of fibrosis and inflammation were significantly elevated after myocardial infarction. Fibrosis, and several cytokines induced by macrophages, can aggravate arrhythmogenic properties.^23^ The results of our study showed significant inverse correlations between inflammatory cytokine release and the CAP‐elicited activation of AMPK signalling. Additionally, our research in LPS‐induced primary macrophages showed that the nuclear translocation of NF‐κB p65 was significantly inhibited by PNU treatment, and suppressing AMPK signalling blunted the observed inhibition of translocation. These suggested that the CAP may have an influence on macrophage polarization through AMPK signalling. Our study suggested that eliciting the CAP could activate AMPK and facilitate the transformation of Ly6C^high^ macrophages into Ly6C^low^ macrophages. Furthermore, eliciting the CAP can influence STAT3 signalling via AMPK signalling. STAT3 signalling is involved in anti‐inflammation and the transformation of macrophages. It is interesting that researchers have two opposing views on the influence of the CAP on the anti‐inflammatory effects of STAT3 signalling pathways, but both viewpoints agree that the STAT3 signalling pathway is anti‐inflammatory and adjusts macrophage polarization.^47^, ^48^, ^49^ Peña et al and Yang et al considered that un‐phosphorylated STAT3 could interfere with the LPS‐induced pro‐inflammatory response via binding of NF‐κB, prevents the activation of NF‐κB.^48^, ^49^ The activation of AMPKα have an inhibitory effect on STAT3.^50^ Our experimental data support the findings above.

In addition, α7‐nAChR agonists can up‐regulate the receptor expression,^51^ We think this may be a mechanism of feedback. The neurovirulence of oxaliplatin decreased the expression of the alpha7 receptor, and the PNU increased the level of the receptor. In our preliminary experiment the alpha7 receptor expression of ICM rats was increased, and the treatment with PNU restored the normal level of the receptor (see supplements, Figure S3). These two opposite effects are both for regulating the abnormal expression of the alpha7 receptor in the pathological condition. They are protective effects and a mechanism of feedback. Furthermore, evidence indicates that α7‐nAChR can desensitize rapidly in response to high agonist concentrations in vitro, which may preclude us from observing the effect of receptor activation.^52^, ^53^ In the normal neuromuscular junction, the delivery and removal of acetylcholine is very rapid so that desensitization is usually not considered to be important. But the process of desensitization is complicated and still worth for further researches. Study the desensitization will lead us to the best dosage of the agonist for most beneficial effect.

## 5 CONCLUSIONS

In this study, we demonstrated that eliciting the CAP can exert protective effects in cardiac ischaemia and ICM‐induced heart failure and ventricular arrhythmia via activation of AMPK signalling. The CAP could be a potential therapeutic for ICM‐induced heart failure and an alternative therapeutic strategy for ICM‐induced ventricular arrhythmia.

## DISCLOSURES

The authors declare that the research was conducted in the absence of any commercial or financial relationships that could be construed as a potential conflict of interest.

## References

[1] Lozano R , Naghavi M , Foreman K , et al. Global and regional mortality from 235 causes of death for 20 age groups in 1990 and 2010: a systematic analysis for the Global Burden of Disease Study 2010. Lancet. 2012;380(9859):2095‐2128. 10.1016/S0140-6736(12)61728-0 23245604 PMC10790329

[2] Wong SC , Fukuchi M , Melnyk P , Rodger I , Giaid A . Induction of cyclooxygenase‐2 and activation of nuclear factor‐kappaB in myocardium of patients with congestive heart failure. Circulation. 1998;98(2):100‐103. PMID: 9679714. 9679714 10.1161/01.cir.98.2.100

[3] Wu SJ , Li YC , Shi ZW , et al. Alteration of cholinergic anti‐inflammatory pathway in rat with ischemic cardiomyopathy‐modified electrophysiological function of heart. J Am Heart Assoc. 2017;6(9). pii: e006510 10.1161/JAHA.117.006510 28928157 PMC5634297

[4] Wang H , Yu M , Ochani M , et al. Nicotinic acetylcholine receptor alpha7 subunit is an essential regulator of inflammation. Nature. 2003;421(6921):384‐388. 10.1038/nature01339 12508119

[5] Saeed RW , Varma S , Peng‐Nemeroff T , et al. Cholinergic stimulation blocks endothelial cell activation and leukocyte recruitment during inflammation. J Exp Med. 2005;201(7):1113‐1123. 10.1084/jem.20040463 15809354 PMC2213139

[6] Zanetti SR , Ziblat A , Torres NI , et al. Expression and functional role of α7 nicotinic receptor in human cytokine‐stimulated natural killer (NK) cells. J Biol Chem. 2016;291(32):16541‐16552. 10.1074/jbc.M115.710574 27284006 PMC4974370

[7] Rothbard JB , Rothbard JJ , Soares L , et al. Identification of a common immune regulatory pathway induced by small heat shock proteins, amyloid fibrils, and nicotine. Proc Natl Acad Sci U S A. 2018;115(27):7081‐7086. 10.1073/pnas.1804599115 29915045 PMC6142248

[8] Leib C , Göser S , Lüthje D , et al. Role of the cholinergic antiinflammatory pathway in murine autoimmune myocarditis. Circ Res. 2011;109(2):130‐140. 10.1161/CIRCRESAHA.111.245563 21597011

[9] Guan YZ , Jin XD , Guan LX , et al. Nicotine inhibits microglial proliferation and is neuroprotective in global ischemia rats. Mol Neurobiol. 2015;51(3):1480‐1488. 10.1007/s12035-014-8825-3 25095782

[10] Cooke AA , Connaughton RM , Lyons CL , et al. Fatty acids and chronic low grade inflammation associated with obesity and the metabolic syndrome. Eur J Pharmacol. 2016;15(785):207‐214. 10.1016/j.ejphar.2016.04.021 27083551

[11] Murphy AM , Lyons CL , Finucane OM , Roche HM . Interactions between differential fatty acids and inflammatory stressors‐impact on metabolic health. Prostaglandins Leukot Essent Fatty Acids. 2015;92:49‐55. 10.1016/j.plefa.2014.05.003 24947613

[12] O'Reilly M , Dillon E , Guo W , et al. High‐density lipoprotein proteomic composition, and not efflux capacity, reflects differential modulation of reverse cholesterol transport by saturated and monounsaturated fat diets. Circulation. 2016;133(19):1838‐1850. 10.1161/CIRCULATIONAHA.115.020278 27081117 PMC6122580

[13] Steinberg GR , Kemp BE . AMPK in health and disease. Physiol Rev. 2009;89(3):1025‐1078. 10.1152/physrev.00011.2008 19584320

[14] Wang Q , Zhang M , Torres G , et al. Metformin suppresses diabetes‐accelerated atherosclerosis via the inhibition of Drp1‐mediated mitochondrial fission. Diabetes. 2017;66(1):193‐205. 10.2337/db16-0915 27737949 PMC5204316

[15] Krawczyk CM , Holowka T , Sun J , et al. Toll‐like receptor‐induced changes in glycolytic metabolism regulate dendritic cell activation. Blood. 2010;115(23):4742‐4749. 10.1182/blood-2009-10-249540 20351312 PMC2890190

[16] Jayasankar V , Woo YJ , Bish LT , et al. Inhibition of matrix metalloproteinase activity by TIMP‐1 gene transfer effectively treats ischemic cardiomyopathy. Circulation. 2004;110(11 Suppl. 1):II180–II186. 10.1161/01.CIR.0000138946.29375.49 15364860

[17] Felker GM , Shaw LK , O'Connor CM . A standardized definition of ischemic cardiomyopathy for use in clinical research. J Am Coll Cardiol. 2002;39(2):210‐218. PMID: 11788209. 11788209 10.1016/s0735-1097(01)01738-7

[18] Bélichard P , Savard P , Cardinal R , et al. Markedly different effects on ventricular remodeling result in a decrease in inducibility of ventricular arrhythmias. J Am Coll Cardiol. 1994;23(2):505‐513. PMID: 8294707. 8294707 10.1016/0735-1097(94)90440-5

[19] Nguyen T , El Salibi E , Rouleau JL . Postinfarction survival and inducibility of ventricular arrhythmias in the spontaneously hypertensive rat: effects of ramipril and hydralazine. Circulation. 1998;98(19):2074‐2080. PMID: 9808607. 9808607 10.1161/01.cir.98.19.2074

[20] Zhang X , Goncalves R , Mosser DM . The isolation and characterization of murine macrophages. Curr Protoc Immunol. 2008;Chapter 14: Unit 14.1. 10.1002/0471142735.im1401s83 PMC2834554 19016445

[21] Kim KH , Lee MS . Autophagy–a key player in cellular and body metabolism. Nat Rev Endocrinol. 2014;10(6):322‐337. 10.1038/nrendo.2014.35 24663220

[22] Nahrendorf M , Swirski FK , Aikawa E , et al. The healing myocardium sequentially mobilizes two monocyte subsets with divergent and complementary functions. J Exp Med. 2007;204(12):3037‐3047. 10.1084/jem.20070885 18025128 PMC2118517

[23] Nguyen TP , Qu Z , Weiss JN . Cardiac fibrosis and arrhythmogenesis: the road to repair is paved with perils. J Mol Cell Cardiol. 2014;70:83‐91. 10.1016/j.yjmcc.2013.10.018 24184999 PMC3995831

[24] Hamid T , Guo SZ , Kingery JR , et al. Cardiomyocyte NF‐κB p65 promotes adverse remodelling, apoptosis, and endoplasmic reticulum stress in heart failure. Cardiovasc Res. 2011;89(1):129‐138. 10.1093/cvr/cvq274 20797985 PMC3002872

[25] Friedman PL , Fenoglio JJ , Wit AL . Time course for reversal of electrophysiological and ultrastructural abnormalities in subendocardial Purkinje fibers surviving extensive myocardial infarction in dogs. Circ Res. 1975;36(1):127‐144. PMID: 1116215. 1116215 10.1161/01.res.36.1.127

[26] Spear JF , Michelson EL , Moore EN . Cellular electrophysiologic characteristics of chronically infarcted myocardium in dogs susceptible to sustained ventricular tachyarrhythmias. J Am Coll Cardiol. 1983;1(4):1099‐1110. PMID: 6833648. 6833648 10.1016/s0735-1097(83)80112-0

[27] Matsushita T , Oyamada M , Fujimoto K , et al. Remodeling of cell‐cell and cell‐extracellular matrix interactions at the border zone of rat myocardial infarcts. Circ Res. 1999;85(11):1046‐1055. PMID: 10571536. 10571536 10.1161/01.res.85.11.1046

[28] Matsushita T , Takamatsu T . Ischaemia‐induced temporal expression of connexin43 in rat heart. Virchows Arch. 1997;431(6):453‐458. PMID: 9428934. 9428934 10.1007/s004280050123

[29] Gutstein DE , Morley GE , Tamaddon H , et al. Conduction slowing and sudden arrhythmic death in mice with cardiac‐restricted inactivation of connexin43. Circ Res. 2001;88(3):333‐339. PMID: 11179202. 11179202 10.1161/01.res.88.3.333 PMC3630465

[30] Lerner DL , Yamada KA , Schuessler RB , Saffitz JE . Accelerated onset and increased incidence of ventricular arrhythmias induced by ischemia in Cx43‐deficient mice. Circulation. 2000;101(5):547‐552. PMID: 10662753. 10662753 10.1161/01.cir.101.5.547

[31] Yao JA , Gutstein DE , Liu F , et al. Cell coupling between ventricular myocyte pairs from connexin43‐deficient murine hearts. Circ Res. 2003;93(8):736‐743. 10.1161/01.RES.0000095977.66660.86 14500334

[32] Niger C , Howell FD , Stains JP . Interleukin‐1beta increases gap junctional communication among synovial fibroblasts via the extracellular‐signal‐regulated kinase pathway. Biol Cell. 2009;102(1):37‐49. 10.1042/BC20090056 19656083 PMC2874634

[33] Janse MJ , Wit AL . Electrophysiological mechanisms of ventricular arrhythmias resulting from myocardial ischemia and infarction. Physiol Rev. 1989;69(4):1049‐1169. 10.1152/physrev.1989.69.4.1049 2678165

[34] Marks AR . Calcium cycling proteins and heart failure: mechanisms and therapeutics. J Clin Invest. 2013;123(1):46‐52. 10.1172/JCI62834 23281409 PMC3533269

[35] Ingwall JS . On the hypothesis that the failing heart is energy starved: lessons learned from the metabolism of ATP and creatine. Curr Hypertens Rep. 2006;8(6):457‐464. PMID: 17087856. 17087856 10.1007/s11906-006-0023-x

[36] Jäger S , Handschin C , St‐Pierre J , Spiegelman BM . AMP‐activated protein kinase (AMPK) action in skeletal muscle via direct phosphorylation of PGC‐1alpha. Proc Natl Acad Sci U S A. 2007;104(29):12017‐12022. 10.1073/pnas.0705070104 17609368 PMC1924552

[37] Iglesias MA , Furler SM , Cooney GJ , et al. AMP‐activated protein kinase activation by AICAR increases both muscle fatty acid and glucose uptake in white muscle of insulin‐resistant rats in vivo. Diabetes. 2004;53(7):1649‐1654. PMID: 15220186. 15220186 10.2337/diabetes.53.7.1649

[38] Sozio MS , Lu C , Zeng Y , et al. Activated AMPK inhibits PPAR‐{alpha} and PPAR‐{gamma} transcriptional activity in hepatoma cells. Am J Physiol Gastrointest Liver Physiol. 2011;301(4):G739‐G747. 10.1152/ajpgi.00161.2011 21700905 PMC3191559

[39] Alesutan I , Voelkl J , Stöckigt F , et al. AMP‐activated protein kinase α1 regulates cardiac gap junction protein connexin 43 and electrical remodeling following pressure overload. Cell Physiol Biochem. 2015;35(1):406‐418. 10.1159/000369706 25591781

[40] Chi Y , Gao K , Li K , et al. Purinergic control of AMPK activation by ATP released through connexin 43 hemichannels ‐ pivotal roles in hemichannel‐mediated cell injury. J Cell Sci. 2014;127(Pt 7):1487‐1499. 10.1242/jcs.139089 24496445

[41] Harada M , Nattel SN , Nattel S . AMP‐activated protein kinase: potential role in cardiac electrophysiology and arrhythmias. Circ Arrhythm Electrophysiol. 2012;5(4):860‐867. 10.1161/CIRCEP.112.972265 22895602

[42] Hulsmans M , Clauss S , Xiao L , et al. Macrophages facilitate electrical conduction in the heart. Cell. 2017;169(3):510‐522.e20. 10.1016/j.cell.2017.03.050 28431249 PMC5474950

[43] Hume DA . Differentiation and heterogeneity in the mononuclear phagocyte system. Mucosal Immunol. 2008;1(6):432‐441. 10.1038/mi.2008.36 19079210

[44] Weidenbusch M , Anders HJ . Tissue microenvironments define and get reinforced by macrophage phenotypes in homeostasis or during inflammation, repair and fibrosis. J Innate Immun. 2012;4(5–6):463‐477. 10.1159/000336717 22507825 PMC6741480

[45] Choo EH , Lee JH , Park EH , et al. Infarcted myocardium‐primed dendritic cells improve remodeling and cardiac function after myocardial infarction by modulating the regulatory T cell and macrophage polarization. Circulation. 2017;135(15):1444‐1457. 10.1161/CIRCULATIONAHA.116.023106 28174192

[46] Jiang W , St‐Pierre S , Roy P , et al. Infiltration of CCR47+Ly6Chigh proinflammatory monocytes and neutrophils into the central nervous system is modulated by nicotinic acetylcholine receptors in a model of multiple sclerosis. J Immunol. 2016;196(5):2095‐2108. 10.4049/jimmunol.1501613 26810225 PMC4760232

[47] Joe Y , Kim HJ , Kim S , et al. Tristetraprolin mediates anti‐inflammatory effects of nicotine in lipopolysaccharide‐stimulated macrophages. J Biol Chem. 2011;286(28):24735‐24742. 10.1074/jbc.M110.204859 21606497 PMC3137049

[48] Peña G , Cai B , Liu J , et al. Unphosphorylated STAT3 modulates alpha 7 nicotinic receptor signaling and cytokine production in sepsis. Eur J Immunol. 2010;40(9):2580‐2589. 10.1002/eji.201040540 20706987 PMC3086065

[49] Yang J , Liao X , Agarwal MK , et al. Unphosphorylated STAT3 accumulates in response to IL‐6 and activates transcription by binding to NFkappaB. Genes Dev. 2007;21(11):1396‐1408. 10.1101/gad.1553707 17510282 PMC1877751

[50] Hattori Y , Hattori K , Hayashi T . Pleiotropic benefits of metformin: macrophage targeting its anti‐inflammatory mechanisms. Diabetes. 2015;64(6):1907‐1909. 10.2337/db15-0090 25999535

[51] Di Cesare ML , Pacini A , Matera C , et al. Involvement of α7 nAChR subtype in rat oxaliplatin‐induced neuropathy: effects of selective activation. Neuropharmacology. 2014;79:37‐48. 10.1016/j.neuropharm.2013.10.034 24225197

[52] Thomsen MS , Mikkelsen JD . The α7 nicotinic acetylcholine receptor ligands methyllycaconitine, NS6740 and GTS‐21 reduce lipopolysaccharide‐induced TNF‐α release from microglia. J Neuroimmunol. 2012;251(1–2):65‐72. 10.1016/j.jneuroim.2012.07.006 22884467

[53] Dani JA , Radcliffe KA , Pidoplichko VI . Variations in desensitization of nicotinic acetylcholine receptors from hippocampus and midbrain dopamine areas. Eur J Pharmacol. 2000;393(1‐3):31‐38. 10770995 10.1016/s0014-2999(00)00003-0

