# Supplementary material for: Eliciting α7‐nAChR exerts cardioprotective effects on ischemic cardiomyopathy via activation of AMPK signalling
Source: J Cell Mol Med. 2019 May 6;23(7):4746–58. doi: 10.1111/jcmm.14363 (PMC6584557; doi:10.1111/jcmm.14363)
Supplement: Supplementary file 1 [file JCMM-23-4746-s001.docx]

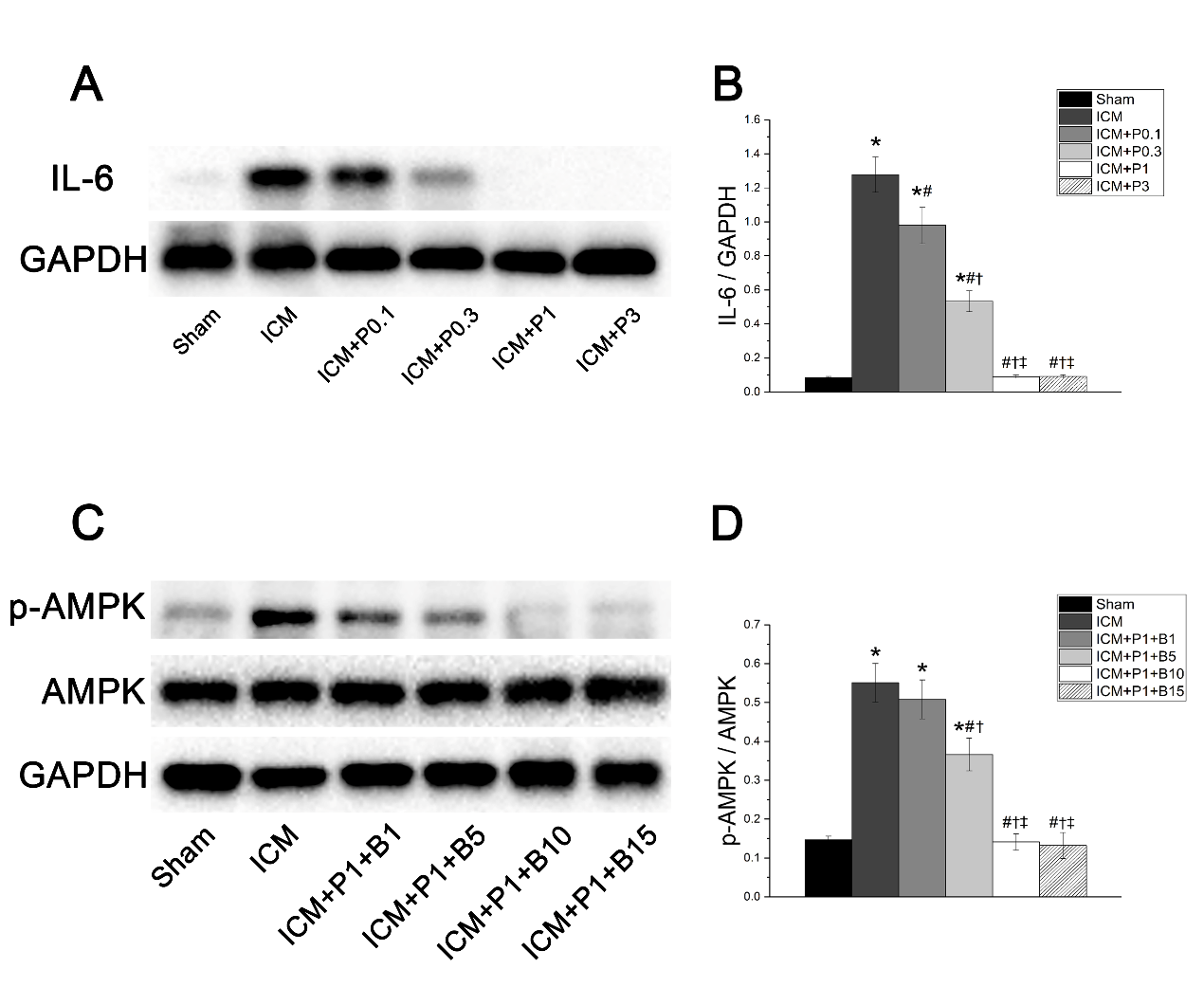


Figure S1. Sham, rats with sham operate. ICM, rats with left anterior descending coronary artery ligation and fed for 4 weeks. P0.1, PNU 0.1mg/kg. P0.3, PNU 0.3mg/kg. P1, PNU 1mg/kg. P3, PNU 3mg/kg. B1, BML 1mg/kg. B5, BML 5mg/kg. B10, BML 10mg/kg. B15, BML 15mg/kg. All PNU and BML were intraperitoneal injection every day for 4 weeks. Every group n=6. The ICM+P1 group was similar to the Sham group and ICM+P3 group (P>0.05). The ICM+P1+B10 group was similar to the Sham group and ICM+P1+B15 group (P>0.05).

*P<0.05, compared with Sham group.

^#^P<0.05, compared with ICM group.

^†^P<0.05, compared with ICM+P0.1 group (B) or compared with ICM+P1+B1 group (D)

^‡^P<0.05, compared with ICM+P0.3 group (B) or compared with ICM+P1+B5 group (D)


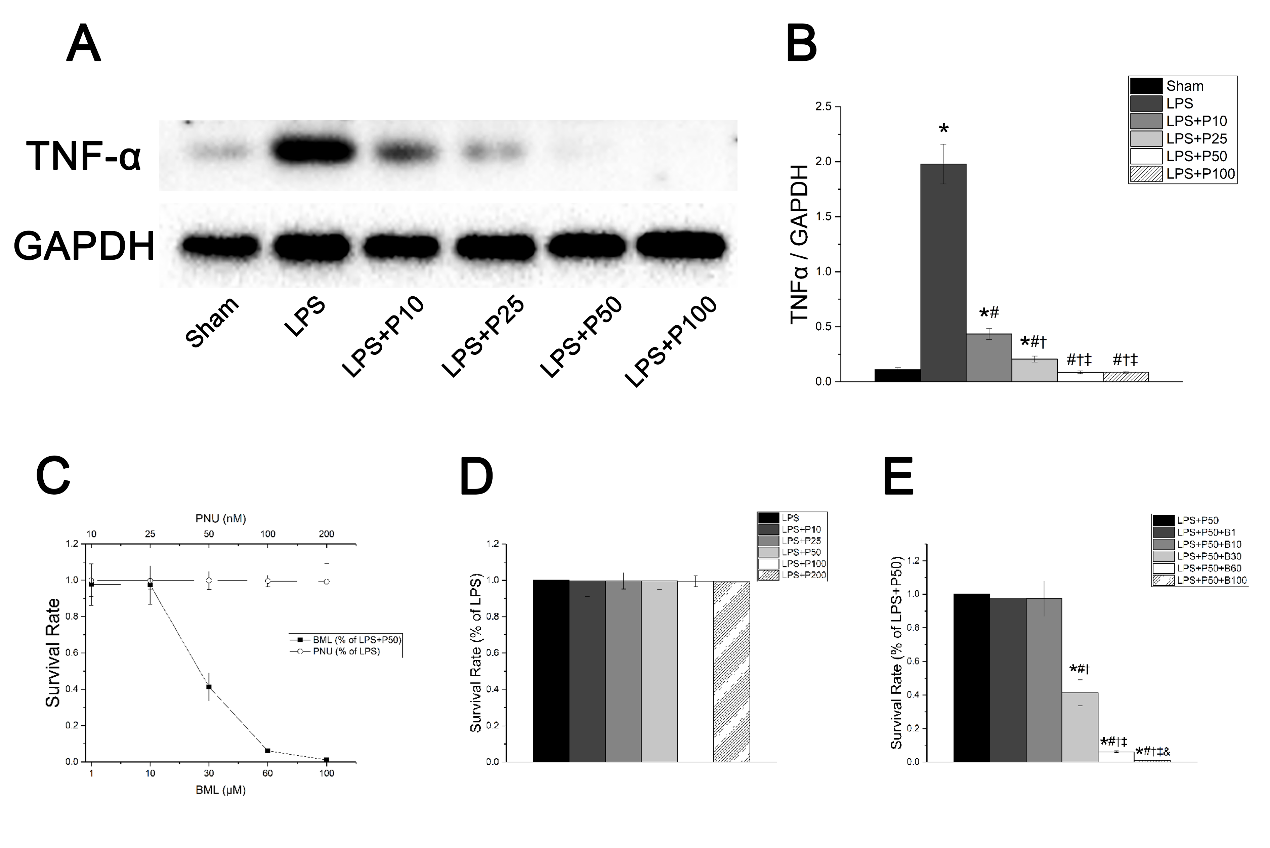


Figure S2. Sham, cells without any special treatment. LPS, cells stimulated with lipopolysaccharide (100 ng/mL). P10, PNU 10nM. P25, PNU 25nM. P50, PNU 50nM. P100, PNU 100nM. P200, PNU 200nM. B1, BML 1μM. B10, BML 10μM. B30, BML 30μM. B60, BML 60μM. B100, BML 100μM. Every group n=6. A to B, The LPS+P50 group was similar to the Sham group and LPS+P100 group (P>0.05). C to E, the survival rate (% of LPS group) of each PNU treatment was similar (D, compare the groups in pairs, P>0.05), and the survival rate (% of LPS+P50 group) among LPS+P50+B10 group, LPS+P50+B1 group and LPS+P50 group were similar (E, compare the groups in pairs, P>0.05).

*P<0.05, compared with Sham group (B) or compared with LPS+P50 group (E).

^#^P<0.05, compared with LPS group (B) or compared with LPS+P50+B1 group (E).

^†^P<0.05, compared with LPS+P10 group (B) or compared with LPS+P50+B10 group (D)

^‡^P<0.05, compared with LPS+P25 group (B) or compared with LPS+P50+B30 group (D)

^&^P<0.05, compared with LPS+P50+B60 group.


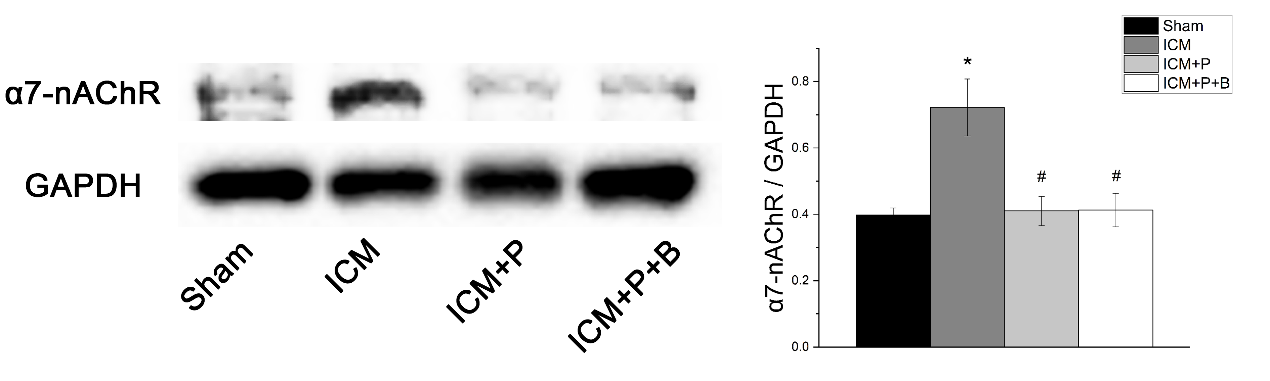


Figure S3. Sham, rats with sham operate. ICM, rats with left anterior descending coronary artery ligation and fed for 4 weeks. P, PNU 1mg/kg intraperitoneal injection every day for 4 weeks. B, BML 10mg/kg intraperitoneal injection every day for 4 weeks. Every group n=6. The ICM+P group was similar to Sham group and ICM+P+B group (P>0.05).

*P<0.05, compared with Sham group.

^#^P<0.05, compared with ICM group.


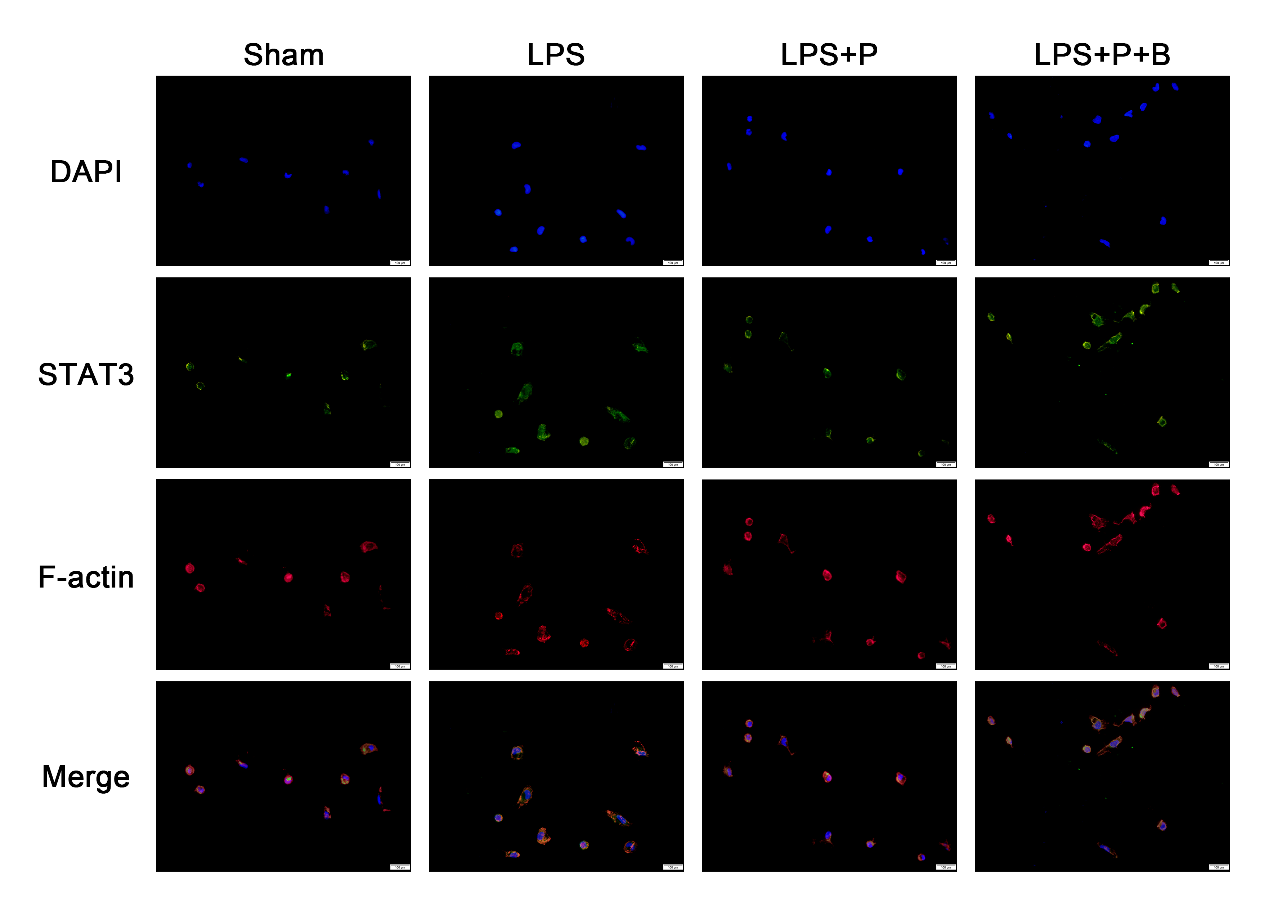


Figure S4. Sham, cells without any special treatment. LPS, cells stimulated with lipopolysaccharide (100 ng/mL). P, PNU 50nM. B, BML 10μM. The PNU treatment suppressed STAT3 activation in LPS-stimulated primary macrophages. BML partly inhibited the effect of PNU on nuclear translocation of LPS-induced STAT3. STAT3 antibody labeled in green with FITC. The nuclei were stained in blue with DAPI. And the cytoskeleton was marked in red with rhodamine phalloidin. The magnification of these representative immunofluorescence images was ×400.
